# A Novel Vaccine Delivery Model of the Apicomplexan *Eimeria tenella* Expressing *Eimeria maxima* Antigen Protects Chickens against Infection of the Two Parasites

**DOI:** 10.3389/fimmu.2017.01982

**Published:** 2018-01-10

**Authors:** Xinming Tang, Xianyong Liu, Guangwen Yin, Jingxia Suo, Geru Tao, Sixin Zhang, Xun Suo

**Affiliations:** ^1^State Key Laboratory of Agrobiotechnology, College of Veterinary Medicine, China Agricultural University, Beijing, China; ^2^Key Laboratory of Animal Epidemiology of the Ministry of Agriculture, College of Veterinary Medicine, China Agricultural University, Beijing, China; ^3^National Animal Protozoa Laboratory, College of Veterinary Medicine, China Agricultural University, Beijing, China; ^4^Engineering Laboratory of Animal Pharmaceuticals, College of Animal Science, Fujian Agriculture and Forestry University, Fuzhou, Fujian, China

**Keywords:** vaccine delivery model, transgenic *Eimeria*, immune mapped protein 1, protective immunity, *Eimeria maxima*

## Abstract

Vaccine delivery is critical in antigen discovery and vaccine efficacy and safety. The diversity of infectious diseases in humans and livestock has required the development of varied delivery vehicles to target different pathogens. In livestock animals, previous strategies for the development of coccidiosis vaccines have encountered several hurdles, limiting the development of multiple species vaccine formulations. Here, we describe a novel vaccine delivery system using transgenic *Eimeria tenella* expressing immunodominant antigens of *Eimeria maxima*. In this delivery system, the immune mapped protein 1 of *E. maxima* (EmIMP1) was delivered by the closely related species of *E. tenella* to the host immune system during the whole endogenous life cycle. The overexpression of the exogenous antigen did not interfere with the reproduction and immunogenicity of transgenic *Eimeria*. After immunization with the transgenic parasite, we detected EmIMP1’s and *E. maxima* oocyst antigens’ specific humoral and cellular immune responses. In particular, we observed partial protection of chickens immunized with transgenic *E. tenella* against subsequent *E. maxima* infections. Our results demonstrate that the transgenic *Eimeria* parasite is an ideal coccidia antigen delivery vehicle and represents a new type of coccidiosis vaccines. In addition, this model could potentially be used in the development of malaria live sporozoite vaccines, in which antigens from different strains can be expressed in the vaccine strain.

## Introduction

Apicomplexan genera parasites that cause serious human, veterinary, or zoonotic diseases include *Plasmodium, Toxoplasma, Eimeria, Neospora*, and *Theileria* (1–4). Chicken coccidiosis caused by the genus *Eimeria* parasites occurs in almost all the poultry farms, results in approximately £2 billion in losses per year and limits the development of the modern poultry industry (5). It has been proposed that the modern poultry industry could have never developed to its present extent without the advent of drugs to control coccidiosis (6). Most drugs are no longer as effective as when they were first introduced due to the development of drug resistance. There is also risk of potential drug residues in poultry meat that threaten food security. Both problems constitute major limitations of anti-coccidiosis drugs (6, 7). Instead of drugs, vaccination with formulations containing either the virulent or the attenuated live parasites has been considered the most efficient method for the protection of breeder and layer flocks from *Eimeria* spp. infection, preventing the presence of drug residues in poultry productions (8, 9). Vaccinated chicks with one *Eimeria* species showed complete defense against subsequent homogeneous infection after self-boosting, but not against heterogeneous parasites (7, 9, 10). These vaccine formulations with *Eimeria* parasites have resulted in commercially available anticoccidial vaccines with live parasites formulations containing multiple species of *Eimeria*. Some require at least three *Eimeria* species (*Eimeria acervulina, Eimeria maxima*, and *Eimeria tenella*) for broilers and five for layers and breeders. However, the requirement of having to simultaneously maintain multiple parasite lines and their production capacity, in addition to economics costs, have been proven to be hurdles to advance the development of these multiple species vaccine formulations (5, 11). Reduction of live parasites formulations, especially of the pathogenic species of the anti-coccidiosis vaccines, can be beneficial in improving animals’ welfare and reducing vaccine production cost.

*Eimeria maxima* is the most immunogenic among the seven species of the *Eimeria* parasites that infect chickens, causing pathological damage in the mild intestine. Infection with as few as five *E. maxima* oocysts can induce complete protective immunity against subsequent homologous challenges (5, 10). The degree of cross-protection among different strains of *E. maxima* is quite variable. In contrast, all six other species of *Eimeria* are perfectly protected against different strains of the same species. Previous studies have shown that just six regions in the *E. maxima* genome were targeted by immunity and two of these have now been interrogated to determine the protective antigen-encoding gene. A new highly protective antigen for *E. maxima*, termed immune mapped protein 1 (IMP1), was identified using genetic mapping techniques (12). Vaccination with the recombinant IMP1 of *E. maxima* by intramuscular injection induces an immune response but provides only partial protection. In addition, none of the vaccine candidates has been tested in commercial applications (5, 11). However, since protective antigens may not be identified until a suitable method of delivery has been developed and a suitable method of delivery may not be recognized until protective antigens have been isolated, the present scenario for the identification of putative immunogenic molecules of *Eimeria* represents a classic “Catch 22” situation (5, 11). We hypothesized that an antigen delivery vehicle carrying protective antigens to target the intestinal mucosal immune system would be the best approach for the development of recombinant anti-coccidiosis vaccines based on one or several immune protective antigens.

*Eimeria tenella*, the model organism to study the biological and immunological characteristics of the apicomplexan parasite, has been considered as vaccine delivery vehicle expressing pathogen antigens. These elicit foreign antigen-specific immune responses in chickens and in mammals (13–15). Since there are another six species of *Eimeria* parasites that infect chicken intestinal mucosa in addition to *E. tenella*, we further hypothesized that *E. tenella* would be the best vaccine delivery vehicle to carry *Eimeria* parasite immunodominant antigens to target the intestinal mucosal immune system to provide protective immunity against subsequent parasites infection. To prove this idea, in this study, we constructed a transgenic *E. tenella* line expressing the immunodominant antigen of *E. maxima* (Et-EmIMP1) and used it as anticoccidial vaccine formulation to investigate its protective immunity against heterogeneous *E. maxima* infection in chickens.

## Materials and Methods

### Ethics Statement

All animal experiments were performed in strict accordance with the China Agricultural University Institutional Animal Care and Use Committee guidelines (CAU20160628-2) and followed the International Guiding Principles for Biomedical Research Involving Animals. Experiments were approved by the Beijing Administration Committee of Laboratory Animals.

### Parasites and Animals

*Eimeria tenella* (XJ strain), *E. maxima* (BJ strain), *Eimeria mitis* (CAU strain), and *E. acervulina* (CAU strain) used in this study were maintained by propagating them in coccidian-free, 2- to 5-week-old Arber Acres broilers. The procedures for collection, purification, and sporulation were carried out as previously described (16).

One-week-old SPF chickens were purchased from Merial Animal Health Co., Ltd. (Beijing, China) and were fed a pathogen-free diet and water *ad libitum*.

### Plasmid Construction

Total RNA was isolated from *E. maxima* sporozoites was isolated using the TRIzol reagent (Invitrogen, USA). cDNA was synthesized through the utilization of random primers and a High Capacity cDNA Reverse Transcription Kit (Applied Biosystems, USA). The open reading frame of *E. maxima* IMP1 was amplified by PCR using IMP1-F/IMP1-R1 primers (Table S1 in Supplementary Material) designed according to the IMP1 sequences (GeneBank Accession number: FN813228.1). Flag-tag was targeted at the 3′ end of IMP1 gene by two rounds overlapping PCR using IMP1-F/IMP1-R2 and IMP1-F/IMP1-R3, respectively (Table S1 in Supplementary Material). After PCR amplification, Nde I and Sac II restriction enzyme sites were introduced into IMP1 fragment. The transfection vector, pSDEP2AIMP1S, was constructed based on pSDEP2ARS (17). Briefly, the ssRFP-His tag fragment of pSDEP2ARS was replaced by IMP1-Flag-tag fragment based on Nde I and Sac II restriction enzymes digestion and DNA ligase ligation. The transfection vector was linearized by SnaB I restriction enzyme for further use.

### Construction of the Transgenic *E. tenella* Line

A restriction enzyme-mediated integration was adapted for the transfection of *E. tenella* sporozoites as previously described (18). Stably transfected *E. tenella* line was obtained by pyrimethamine selection combined with fluorescence activated cell sorting (Table S2 in Supplementary Material). Western blot and indirect immunofluorescent assay (IFA) were conducted to confirm foreign antigens expression and distribution in transgenic parasites based on the previous described protocols, respectively (15). Sporozoites were extracted from fresh collected sporulated oocysts. First and secondary schizonts and gametocytes were collected from variable dose EmIMP1 (5 × 10^7^, 1 × 10^7^, and 1 × 10^3^ oocysts, respectively) infected chickens at 3, 5, and 7 dpi using the previous methods (19). Soluble antigens of sporozoites, first-generation schizonts, second-generation schizonts, gametocytes, and unsporulated oocysts were resolved by SDS-PAGE and immunoblot analysis following standard protocols with mouse anti-flag monoclonal antibody and HRP-conjugated goat anti-mouse IgG (Proteintech, USA) as primary and secondary antibody, respectively. Poly-antibodies against *E. tenella* GAPDH and enhanced yellow fluorescent protein (EYFP) served as controls.

Indirect immunofluorescent assays were conducted to detect the distribution and relative expression level of foreign antigen. The primary and secondary antibody were mouse anti-flag monoclonal antibody and Cy3-conjugated goat anti-mouse IgG (Proteintech, USA). Poly-antibodies against endogenous IMP1 of *E. tenella* served as control.

### Quantification of Transgenic Parasite Replication

Three groups of four inbred SPF chickens were inoculated with 100 sporulated wild-type *E. tenella*, EtER (transgene control) and Et-EmIMP1 oocysts at 1 week of age. The output of oocysts of the transgenic lines and wild-type parasite in chickens was measured daily by using McMaster egg counting chamber between 4 and 14 days’ postinoculation, respectively (20).

### Observation of EYFP Expression in the Endogenous Development of the Transgenic Parasites

Six groups of two inbred SPF chickens were inoculated with 5 × 10^7^, 1 × 10^7^, 1 × 10^6^, 1 × 10^5^, 1 × 10^4^, and 1 × 10^3^ sporulated Et-EmIMP1 oocysts at 1 week of age. Chickens were restricted to feed to reduce the amount of cecum. For each infection dose groups, chickens were euthanized every 24 h for necropsy. The cecum tissue was washed three times using cold PBS. Fresh smears were made from pieces of tissues from the mucous membrane of the cecum and visualized using a confocal laser scanning microscope (SP5, Leica, Germany) for the detection of EYFP-expressing parasites.

### Vaccination and Challenge Infection

Four groups of six inbred SPF chickens were either left naïve or were immunized by infection with 200 sporulated wild-type *E. tenella*, EtER, and Et-EmIMP1 oocysts at 1 week of age. Secondary immunization was administrated at 2-week intervals with 1,000 oocysts as immunization dosage for each bird. All chickens were housed in the same conditions of temperature and humidity and fed a coccidian-free diet and water *ad libitum*. Serum was collected when chickens were 1, 3, and 5 weeks old and stored at −20°C until use. Challenge infections with wild-type *E. tenella* (50,000 oocysts) or *E. maxima* (50 oocysts) were conducted 2 weeks after the last immunization (at 5 weeks of the chicken age). Oocyst output and body weight gain of the chickens were evaluated after challenge infection.

### ELISA and ELISPOT

ELISA was conducted as previously described (15, 21). Briefly, 5 µg/ml *E. tenella* or *E. maxima* oocysts antigens or 1 µg/ml recombinant EmIMP1 were coated onto the individual wells of the plates followed by a reaction with serum (1:100) and collected when chicken were 1, 3, and 5 weeks old. The secondary antibody used in this experiment was the HRP-conjugated goat anti-chicken IgY Fc fragment (Bethyl Laboratories, Inc.). Foreign antigen and parasites specific cellular immune responses revealed by IFN-γ secreting cells present in peripheral blood mononuclear cells (PBMCs) after immunization with the transgenic parasite or its wild-type parasite were evaluated by ELISPOT following established protocols in our laboratory (22). Briefly, 10^6^ PBMCs from the naïve, the wild-type *E. tenella*, EtER, and the Et-EmIMP1 oocysts immunized birds were stimulated with 10 µl PBS, 10 µg *E. tenella* or *E. maxima* oocysts antigen or 2 µg recombinant EmIMP1, and 10 µl PMA plus ionomycin (10 ng/ml PMA plus ionomycin 5 µg/ml), respectively. The spots in which IFN-γ secretion lymphocytes were present were detected after 24 h stimulation.

### Floor Pen Test

Groups of 12 inbred SPF chickens were either left naïve (Ctrl) or were immunized by infection with 200 sporulated wild-type *E. tenella* (WT), EtER, and Et-EmIMP1 oocysts at 1 week of age. Chickens were housed in the same conditions of temperature and humidity. New litter of chopped straw was spread over cages’ bottom 5 cm, and chickens were fed a coccidian-free diet and water *ad libitum*. Six chickens of each group were removed to new cages and challenged with *E. maxima* (50 oocysts/bird) at 14 and 28 dpi, respectively. Oocyst outputs were evaluated after each challenge infection.

To test the species-specific protective immunity elicited by Et-EmIMP1, groups of 12 inbred SPF chickens were either left naïve (Ctrl) or were immunized by infection with 200 sporulated wild-type *E. tenella* (WT), Et-EmIMP1 oocysts at 1 week of age. Chickens were housed in the same conditions of temperature and humidity and fed a coccidian-free diet and water *ad libitum*, while all the chickens were infected with *E. acervulina* or *E. mitis* (50 oocysts/bird) at 14 and 28 dpi.

### Statistical Analysis

GraphPad Prism 6.01 (GraphPad Software) was used for statistical analysis. Differences between control and treated groups were analyzed using SPSS 12.0 (SPSS Institute Inc.). Differences in the experimental treatments were tested using Duncan’s Multiple Range Test following ANOVA with significance reported at *p* ≤ 0.05.

## Results

### Construction of Transgenic *E. tenella* Line Expressing *E. maxima* IMP1 (Et-EmIMP1)

**Figure 1:**
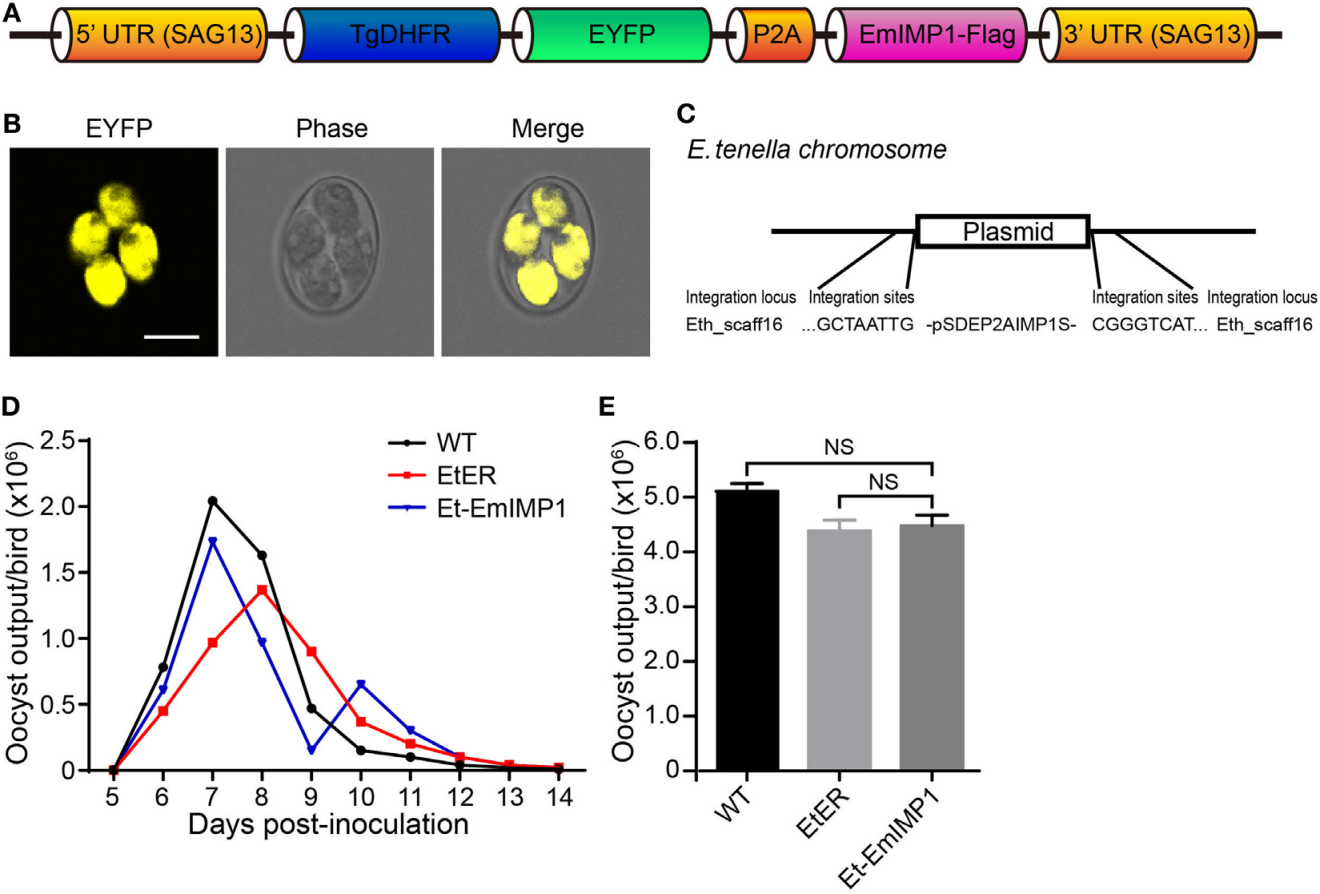
Construction of transgenic *Eimeria tenella* expressing immune mapped protein 1 of *E. maxima* (EmIMP1) and its reproduction. **(A)** EmIMP1 with a flag-tag was co-expressed with TgDHFR-EYFP and linked by P2A in the single expression cassette. **(B)** Stably transfected EmIMP1 expressing reporter enhanced yellow fluorescent protein (EYFP) in its sporulated stage. Bar = 10 μm. **(C)** The exogenous DNA was integrated into Eth_scaff16 according to its flanking sequences obtained by genome walking technique. **(D)** Comparison of oocyst shedding patterns of Et-EmIMP1, its wild type (WT), and the transgenic manipulation control (EtER). The output of oocyst from the three *E. tenella* lines in the parasite infected chickens was measured daily between 5 and 14 days postinoculation (*n* = 3). **(E)** The reproduction of Et-EmIMP1 after inoculation was similar to its wild type and transgenic manipulation control (EtER), as measured by their total oocyst output. NS, not significant. Each value represents the mean ± SD of three birds.

Techniques for the selection of stably transfected *Eimeria* parasites have been successfully developed in the last decade (23–25). To improve the transfection efficiency, here we constructed a relatively short plasmid co-expressing the reporter and target genes (immune mapped protein 1 of *E. maxima*, EmIMP1) mediated by P2A sequence in a single expression cassette plasmid [Figure 1A; Table S1 in Supplementary Material; (17)]. We obtained the stably transfected parasite (Et-EmIMP1) expressing the reporter gene after an exhaustive selection *in vivo* (Figure 1B; Table S2 in Supplementary Material) and confirmed that the exogenous plasmid was integrated into the Eth_scaff 16 locus of the Et-EmIMP1 genome (Figure 1C). We also analyzed the oocysts shedding pattern and total oocyst output of the chickens after inoculation with Et-EmIMP1 and its wild type to reveal the reproduction of the parasites affected by the transgenic manipulation. We found similar oocyst shedding patterns as well as total oocyst output for Et-EmIMP1 and its wild type (Figures 1D,E). The peak corresponding to the maximum oocyst output for the transgenic manipulation control [EtER, a transgenic *E. tenella* line described previously (17)] showed a 24-h delay respect to the wild type (WT) and Et-EmIMP1 (Figure 1D). These data demonstrated that we obtained a pure transgenic *Eimeria* line to investigate its immunogenicity.

### EmIMP1 Was Highly Expressed in the Diverse Endogenous Developmental Stages

**Figure 2:**
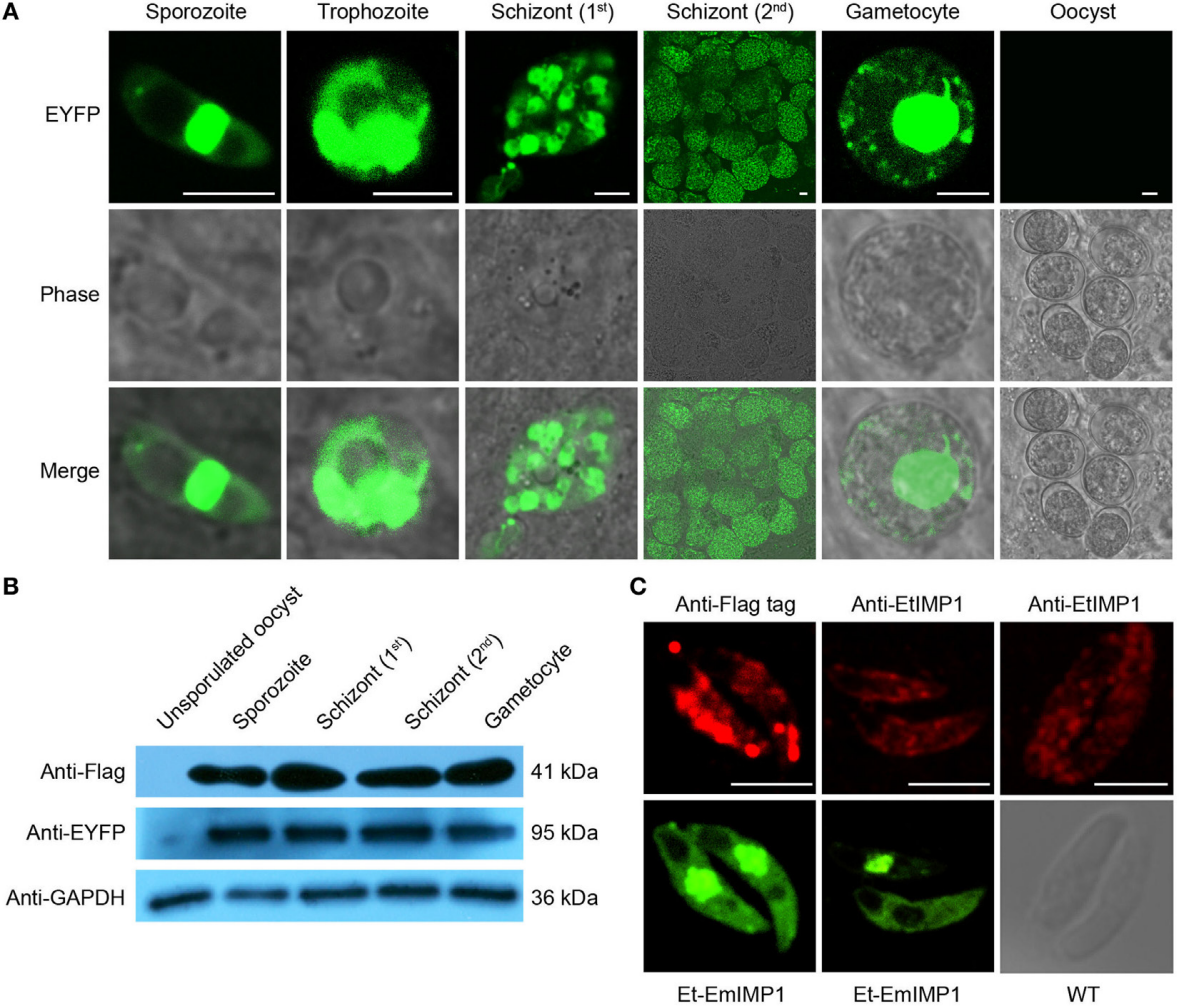
Immune mapped protein 1 of *E. maxima* (EmIMP1) was expressed by Et-EmIMP1 in its diverse endogenous developmental stages. **(A)** Enhanced yellow fluorescent protein (EYFP) was expressed during the whole life cycle of Et-EmIMP1 except during its unsporulated oocyst stage. Dynamic EYFP expression was observed from cecum smear every 24 h after inoculation. Bar = 5 μm. **(B)** Validation of EmIMP1 expression with western blotting. Parasites from different stages (sporozoite, first-generation schizont, and second-generation schizont stages) were immunoblotted with mouse anti-flag-tag monoclonal antibody, while the mouse anti-EtGAPDH polyclonal antibody served as loading control. **(C)** The distribution of EmIMP1 in transgenic sporozoites analyzed by immunofluorescent assay with mouse anti-flag-tag monoclonal antibody. Wild-type sporozoites were used as endogenous immune mapped protein 1 (IMP1) control. Bar = 5 µm.

The schizogony stages are considered to be the most immunogenic of the diverse endogenous developmental stages undergone by eimerian coccidian (10). To observe the EmIMP1 expression pattern during the complex life cycle of the transgenic parasite, we first confirmed that the co-expressing reporter gene was continuously expressed in the diverse endogenous developmental stages of Et-EmIMP1 as sporozoite, trophozoite, schizont, and gametocyte, but not in the unsporulated oocyst stage (Figure 2A). These results suggested that EmIMP1 was expressed in the whole life cycle of Et-EmIMP1, as both EYFP and EmIMP1 were controlled by one set of regulatory elements (17). To further confirm the expression pattern, we conducted an immunoblot assay. Consistent with the expression of EYFP, EmIMP1 was highly expressed in the diverse endogenous developmental stages of the transgenic parasite, except in the unsporulated oocyst stage (Figure 2B).

Homologs of the IMP1 gene can be readily identified in eimerian and non-eimerian apicomplexan parasites, and these may also be candidate protective antigens (12, 26, 27). The IMP1 of *E. tenella* is an immunodominant antigen that elicits partial protective immunity against *E. tenella* infection (28). To determine whether the exogenous EmIMP1 with such high level of expression would interfere with the endogenous EtIMP1, we analyzed the expression and distribution of EmIMP1 and endogenous EtIMP1 for the transgenic parasite. We found that EmIMP1 was expressed in the whole sporozoites of Et-EmIMP1 except in the refractile body (Figure 2C). The endogenous EtIMP1 was expressed mainly on the cell surface of Et-EmIMP1 sporozoites (Figure 2C), as reported in previous experiments with transient transfection of *E. tenella* sporozoites with EtIMP1 tagged with red fluorescent protein (28). In general, EmIMP1 was continuously highly expressed during the whole life cycle of Et-EmIMP1 and did not interfere with the endogenous EtIMP1.

### Et-EmIMP1 Protects Chickens from Parental Parasite Infection

**Figure 3:**
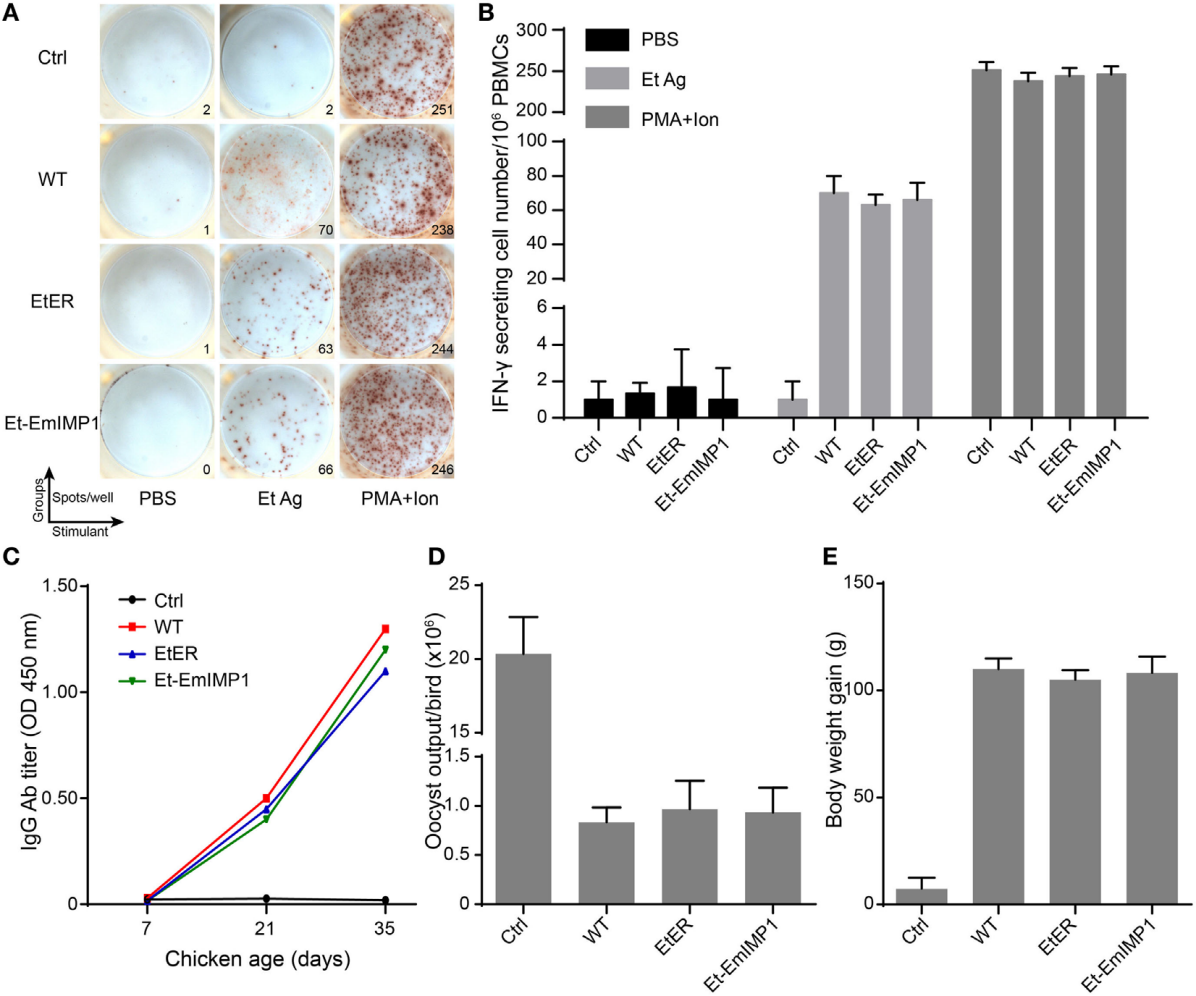
Vaccination with Et-EmIMP1 protected chickens from wild-type *Eimeria tenella* infection. **(A)** Et-EmIMP1 with the immunogenicity that stimulated cellular immune responses as its wild type. Peripheral blood mononuclear cells (PBMCs) from naïve (Ctrl), wild-type *E. tenella* (WT), EtER, and Et-EmIMP1 immunized birds were stimulated with PBS, *E. tenella* oocyst antigens (Et Ag), and PMA plus ionomycin (PMA + ion, positive control). The number of IFN-γ secretion lymphocytes (spots) was measured as described in Section “Materials and Methods.” **(B)** The mean number of Et Ag-specific IFN-γ secretion lymphocytes in PBMCs in Et-EmIMP1 and its wild-type immunized birds showed no significant difference (*n* = 3). **(C)** The Et Ag-specific antibody titer increased after primary (21 days) and secondary (35 days) immunization with Et-EmIMP1 as its wild type. **(D)** Oocyst output after challenge with wild type in the chickens immunized with or without Et-EmIMP1 or its wild type. **(E)** Body weight gain of chickens after 10 days from challenge infection (*n* = 6). Each value represents the mean ± SD of six birds.

Most *Eimeria* parasites are immunogenic pathogens that elicit systemic and mucous immunity against parental parasite reinfection (29). With few exceptions, transgenic manipulation of *Eimeria* parasites expressing molecules not relevant for the immune response did not affect their immunogenicity (25, 30). To evaluate the immunogenicity of our transgenic population expressing high levels of immune molecules compared with its parental strain, we first investigated the cell-mediated immune response stimulated by Et-EmIMP1. This response is revealed by the ratio of parasites’ antigen-specific IFN-γ secreting cells in PBMCs after immunization, as they play a major role in protecting the host from *Eimeria* parasite reinfection (31). We found no significant difference between Et-EmIMP1, its wild-type immunized birds or the transgenic control (EtER) for the *E. tenella* oocyst antigens (Et Ag)-specific in IFN-γ secreting cells in PBMCs (Figures 3A,B). Humoral immunity also contributes to provide additional immunity against *Eimeria* parasites infection, especially against *E. maxima* infection (32). As expected for a cell-mediated immune response, we found that Et-EmIMP1 did not reduce antibody production after immunization (Figure 3C).

To assess the protective efficacy of Et-EmIMP1 as an avian coccidiosis vaccine strain against its parental parasites infection, we infected Et-EmIMP1 immunized birds by oral inoculation with wild-type *E. tenella*. We found there was no oocyst output neither in Et-EmIMP1 nor in its wild-type immunized birds when the challenge dose was 10,000 oocysts (data not shown). When the challenge dose was 50,000 *E. tenella* oocysts, the oocyst output was significantly reduced compared to naïve chickens (Figure 3D). With reduced oocyst output, both Et-EmIMP1 and wild-type parasite immunized birds gained much more body weight (Figure 3E). Taken together, these results clearly show that Et-EmIMP1 maintained the immunogenicity of its wild-type parasite and indicate that a coccidiosis vaccine formulation will protect chickens against *E. tenella* infection.

### Et-EmIMP1 Elicits EmIMP1-Specific Immune Responses That Protects Chickens from *E. maxima* Infection

**Figure 4:**
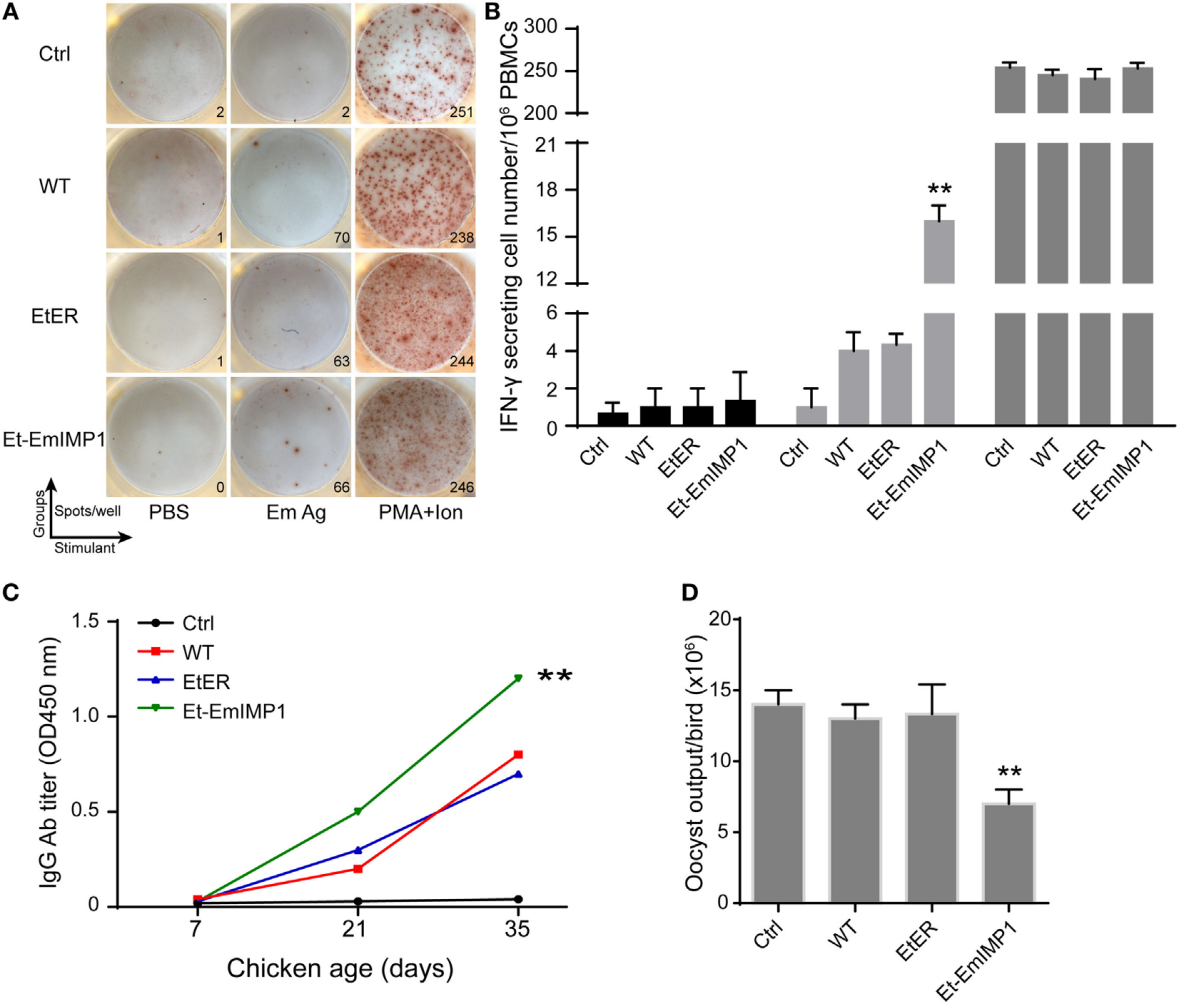
Vaccination with Et-EmIMP1 elicited *Eimeria maxima*-specific immune responses that partly protected chickens from *E. maxima* infection. **(A)** The number of IFN-γ secretion lymphocytes (spots) in peripheral blood mononuclear cells (PBMCs) from naïve (Ctrl), wild-type *Eimeria tenella* (WT), EtER, and Et-EmIMP1 immunized birds after stimulation with PBS, *E. maxima* oocyst antigens (Em Ag), and PMA plus ionomycin (PMA + ion). **(B)** Mean number of Em Ag-specific IFN-γ secretion lymphocytes in PBMCs in Et-EmIMP1 immunized chickens (*n* = 3). **(C)** Em Ag-specific antibody titer increased after primary (21 days) and secondary (35 days) immunization with Et-EmIMP1 and was significantly higher than its wild type. **(D)** Oocyst output after challenge with *E. maxima* in the chickens immunized with or without Et-EmIMP1 or its wild type (*n* = 6).

Vaccination with live parasites (attenuated or un-attenuated strains) is the most powerful way to control coccidiosis in chickens (5, 6, 10). Commercial vaccines against coccidiosis based on live parasites contain multiple species, as there is no cross-protection stimulated by a single parasite. The results presented above showed that Et-EmIMP1 maintained its paternal strain’s immunogenicity protecting chickens from *E. tenella* infection. As next step, we evaluated the EmIMP1 and *E. maxima*-specific immune responses elicited by Et-EmIMP1 as well as the protective efficacy against *E. maxima* infection in chickens. We found that Et-EmIMP1 elicited foreign antigen-specific cellular and humoral immune responses revealed by the number of EmIMP1-specific IFN-γ secreting cells (Figure S1A in Supplementary Material) and antibody production (Figure S1B in Supplementary Material). Immunities against the whole parasite antigens of *E. maxima* were also detected in Et-EmIMP1 immunized chickens, compared to both naïve and WT parasite immunized birds (Figures 4A–C). To test the protective efficacy of these immunities against *E. maxima* infection, we infected the chickens immunized with or without Et-EmIMP1 or its wild-type parasite with 50 *E. maxima* oocysts at 35 dpi and analyzed the *E. maxima* oocyst production. We found significantly reduced oocyst production of Et-EmIMP1 immunized birds’ respect to the wild-type and transfection control groups (Figure 4D). Our results also demonstrated the well-established paradigm indicating that there is no cross-protective immunity between different *Eimeria* species, as the same oocyst output was detected in the naïve and wild-type *E. tenella* immunized birds after *E. maxima* infection (Figure 4D).

Floor pen test is an effective intermediate step in the process of translating an anticoccidial vaccine from the laboratory to commercial trials. We vaccinated 1-week-old SPF chickens with 200 Et-EmIMP1 oocysts or its wild type. The chickens were fed in the cages with litter of chopped straw. Half of the chickens of each group were removed to new cages and infected with 50 *E. maxima* oocysts at 14 and 28 dpi, respectively. We found that protective immunity against *E. maxima* infection was established as early as 14 dpi (Table 1). The immunity was enhanced after self-boosting induced by the offspring oocysts in the litter, as the oocyst production after *E. maxima* infection of Et-EmIMP1 immunized birds was nearly half of the naïve and wild-type parasite immunized birds at 14 dpi and was reduced to nearly 1/3 of the original at 28 dpi (Table 1).

**Table 1:** Oocyst output following challenge infection with *Eimeria maxima* in birds vaccinated with or without Et-EmIMP1 or its wild type.

Groups	Immunization dosage	Oocyst output/bird (×10^6^)	
		14 days after challenge	28 days after challenge
Ctrl	–	11.23 ± 0.35^a^	14.37 ± 0.25^a^
WT	200	12.14 ± 0.40^a^	14.11 ± 0.30^a^
EtER	200	11.78 ± 0.30^a^	13.73 ± 0.27^a^
Et-EmIMP1	200	5.20 ± 0.27^b^	3.60 ± 0.24^b^

*Each value represents the mean ± SD of three independent immunization/challenge trials. For each column (14 or 28 days), values between groups with different superscripts (a and b) are significantly different (*p* < 0.05) according to the Duncan’s multiple range test*.

**Figure 5:**
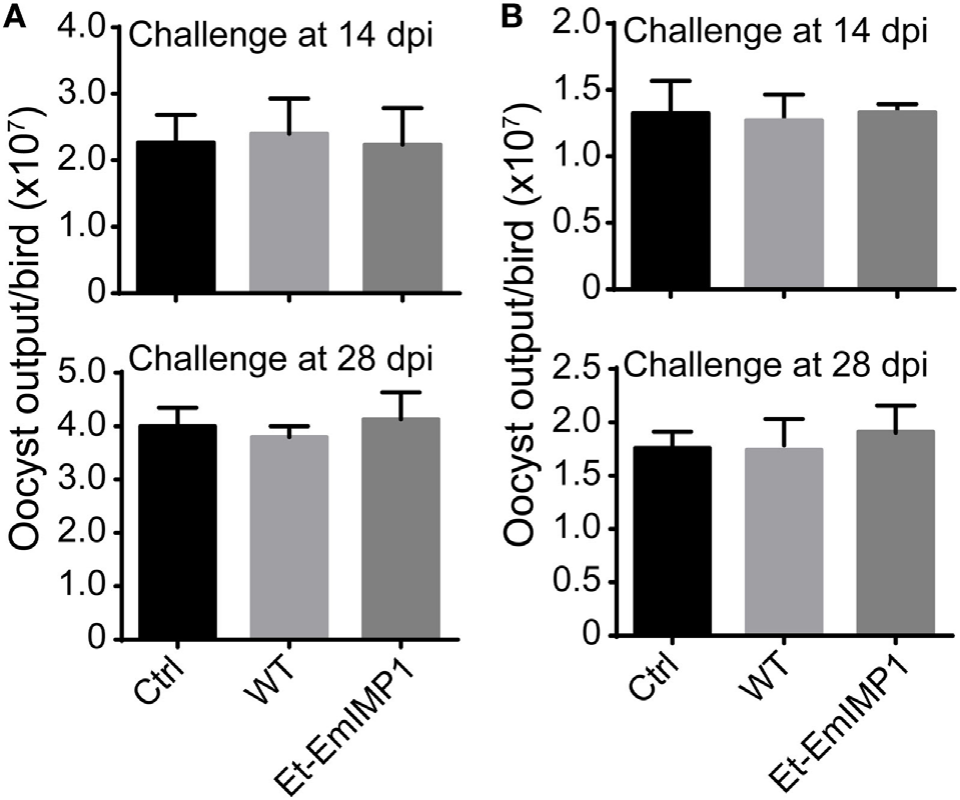
Vaccination with Et-EmIMP1 did not protect chickens from *Eimeria acervulina* and *Eimeria mitis* infection. Oocyst output was similar after challenge with *E. acervulina* **(A)** *or E. mitis* **(B)** in the chickens immunized with or without Et-EmIMP1 or its wild type (*n* = 6).

Immune mapped protein 1 has been identified as immunoprotective antigen from other apicomplexan parasites, such as *E. tenella, Toxoplasma*, and *Neospora* (26–28). We tested for the possible cross-protective immunity elicited by the overexpressed EmIMP1 against other *Eimeria* spp., as it is known that several epitopes are conserved among the seven *Eimeria* spp. in chickens (Figure S2 in Supplementary Material). We infected the birds with Et-EmIMP1 or wild-type parasite immunization with 50 *E. acervulina* or *E. mitis* oocysts and found that at 28 dpi there was no significant difference in the *E. acervulina* (Figure 5A) and *E. mitis* (Figure 5B) production between immunized and naïve birds. These results demonstrated that protective immunity elicited by EmIMP1 is species faithful and only protected chickens against *E. maxima* infection but not against the other six species.

## Discussion

In the present study, we demonstrate that transgenic *Eimeria* parasites used as vaccine delivery model expressing antigen of its affinis species effectively elicited long periods of foreign pathogen-specific protective immune responses, protecting the host against challenges with wild-type paternal and its affinis species. These promising results indicate that transgenic *E. tenella* expressing immunodominant antigens of other *Eimeria* parasites, such as *E. maxima*, is a new and more competitive anticoccidial vaccine strain than traditional ones for the following reasons: (1) the use of strains of *E. maxima* is not a requirement when immunizing with transgenic *E. tenella*, thus saving in vaccine production costs (7, 33); (2) it eliminates the pathological adverse reaction caused by vaccination with *E. maxima*; (3) it reduces the “side effects” caused by “self-boosting” immunization because of contact with the offspring oocysts lacking the *E. maxima* oocysts in the litter (33); and (4) protective immunities are self-boosted during reinfection of the transgenic parasites from the offspring (6, 33).

The dose of oocysts intended for each bird varies between vaccine formulations but generally is around 50–100 oocysts for highly immunogenic species such as *E. maxima* (5, 11). The period of establishment of solid immunity also varies between vaccine formulations but generally is around 2–3 weeks for highly immunogenic species such as *E. maxima* and 3–5 weeks for species such as *E. acervulina* and *E. tenella* (10). In contrast, in our study, protective immunity against *E. maxima* infection was established as early as 14 days after vaccination with Et-EmIMP1 (Table 1). Thus, the immunity provided by the transgenic parasites is sufficient to protect chickens against substantial *E. maxima* infection because early infection from the environment is equivalent to boosting immunization in transgenic parasite immunized chickens. The time point of solid immunity against *E. maxima* infection elicited by transgenic *Eimeria* parasites will be delayed by 1–2 weeks compared to *E. maxima*. This is still acceptable and satisfactory as middle immunogenic species such as *E. acervulina* and *E. tenella* need more than 1 or 2 weeks than *E. maxima* to build solid immunity (5, 9–11).

Live vaccines, based on wild-type parasites, have been available to the poultry industry for around 60 years due to its effective immunities against challenge with homologous parasites. A crucial aspect for the success of this type of vaccines is the method by which they are delivered. Any uneven uptake of vaccinal oocysts by individual birds within a large flock can lead to a series of asynchronous infections during the first 3–5 weeks and even cause coccidiosis outbreaks (5). Live-attenuated vaccines contain the precocious lines of *Eimeria* that confer a marked attenuation of virulence in comparison to the wild-type strains. The immunogenicity of the precocious line generally remains similar to that of their wild-type parents (34). Immunization with live oocyst formulation vaccines can be achieved by *in ovo* injection into the embyronating egg (35). This later technique allows the delivery of new types of anticoccidial and other poultry vaccines, but the mechanism by which the oocysts, when injected into the eggs, are able to establish a clear infection in the gut of the developing embryo is not very clear. As with other methods of vaccination, secondary self-boosting immunization from the offspring on the litter is necessary to establish the protective immunity.

Many techniques have been previously used to identify protective antigens of apicomplexan parasites with the objective of developing subunit vaccines, including coccidiosis vaccines. Immunizing hens with affinity-purified antigens from the wall forming bodies of macrogametocytes of *E. maxima* is the only technique with a subunit vaccine currently employed against any protozoan parasite (36, 37). Maternal antibodies pass *via* the egg to the newly hatched chick and provide passive protection of limited duration (5, 6, 10). Immunizing chicks with the recombinant antigens of eimerian parasites, including IMP1, AMA1, profilin, etc., produced by *Escherichia coli* or DNA vaccines also provides partial protection (1, 12, 38, 39). To improve the immunogenicity of the subunit vaccines, various types of molecular adjuvants, such as toll-like receptor ligand or co-stimulators of T cells and cytokines, were introduced by fusing expression with the antigens or co-immunizing with them (40–42). Although enhanced immune responses were detected after immunization that was two to three times higher using subunit vaccines together with the adjuvants, they can hardly protect chickens from subsequent infections of *Eimeria* parasites (22, 43). For this reason, the vaccine delivery vehicle in which *Eimeria* parasites release the antigens as the parasites infect the hosts constitute a breakthrough technique, but it also provides a new direction for the development of recombinant vaccines in livestock and possibly even in humans.

Immunity to *Eimeria* is complex and multifactorial. Anti-CD4 depletion studies in chickens demonstrated that CD4+ but not CD8+ cells were essential for resistance to primary infection with *E. tenella* but not *E. acervulina* (44). The major mediator of immune-mediated resistance to primary infection and parasites killing is IFN-γ, although the source of effective IFN-γ differs between different *Eimeria* species (45, 46). Studies with B cell-deficient mice (47) and bursectomised chickens (48) suggest that B cells play a minor role during *Eimeria* parasite primary infection and are not essential for expression of complete immunity to re-challenge infection, although specific immunoglobulin (IgM, IgG, and IgA) is induced by infection.

In conclusion, the transgenic *Eimeria* parasite expressing immunodominant antigens of other *Eimeria* species provides a new strategy for the development of coccidiosis vaccines. We are convinced that our strategy can be extended to the development of *E. tenella* as a vector carrying the immunodominant antigens of *Eimeria necatrix*, reducing the need for formulations of *E. necatrix* or other pathogenic *Eimeria* species (49). The most promising coccidiosis vaccines will be the ones containing only one species of transgenic *Eimeria* while providing protective immunity against all the *Eimeria* parasites infections. This proposed strategy relies on the use of multiple techniques, including considerable use of molecular biology techniques to identify antigens capable of inducing an immune response. Our results have important implications for the development of live sporozoite malaria vaccines in which antigens from different strains can be expressed in the vaccine strain.

## Ethics Statement

All animal experiments were performed in strict accordance with the China Agricultural University Institutional Animal Care and Use Committee guidelines (CAU20160628-2) and followed the International Guiding Principles for Biomedical Research Involving Animals. Experiments were approved by the Beijing Administration Committee of Laboratory Animals.

## Author Contributions

XT and XS conceived and designed this study and analyzed the data. XT carried out the experiments and drafted the manuscripts. XL, GY, JS, GT, and SZ contributed to help the statistical analysis and help to draft the manuscripts. XS and XL supervised the study implementation and revised the manuscript. All authors read and approved the final version of the manuscript.

## Conflict of Interest Statement

The authors declare that the research was conducted in the absence of any commercial or financial relationships that could be construed as a potential conflict of interest.

## Supplementary Material

Figure S1 Vaccination with Et-EmIMP1 elicited EmIMP1-specific immune responses. **(A)** Mean number of EmIMP1-specific IFN-γ secretion lymphocytes in peripheral blood mononuclear cells (PBMCs) in Et-EmIMP1 immunized chickens (*n* = 3). **(B)** EmIMP1-specific antibody titer increased after primary (21 days) and secondary (35 days) immunization with Et-EmIMP1 significantly higher than its wild type. Each value represents the mean ± SD of three birds. **p* < 0.05; ***p* < 0.01.

Figure S2 Multiple alignment of immune mapped protein 1 (IMP1) of chicken coccidia. Immune mapped protein 1 of *E. maxima* (EmIMP1)’s gene number is given in the Section “Materials and Methods.” GeneDB’s gene numbers of IMP1 of *Eimeria tenella, Eimeria acervulina, Eimeria necatrix, Eimeria mitis, Eimeria brunette, and Eimeria praecox* were ETH_00030475, EAH_00011210, ENH_00036180, EMH_0033960, EBH_0052010, and EPH_0045280, respectively.
