# Supplementary figures and images for: A Novel Vaccine Delivery Model of the Apicomplexan Eimeria tenella Expressing Eimeria maxima Antigen Protects Chickens against Infection of the Two Parasites
Source: Front Immunol. 2018 Jan 10;8:1982. doi: 10.3389/fimmu.2017.01982 (PMC5767589; doi:10.3389/fimmu.2017.01982)

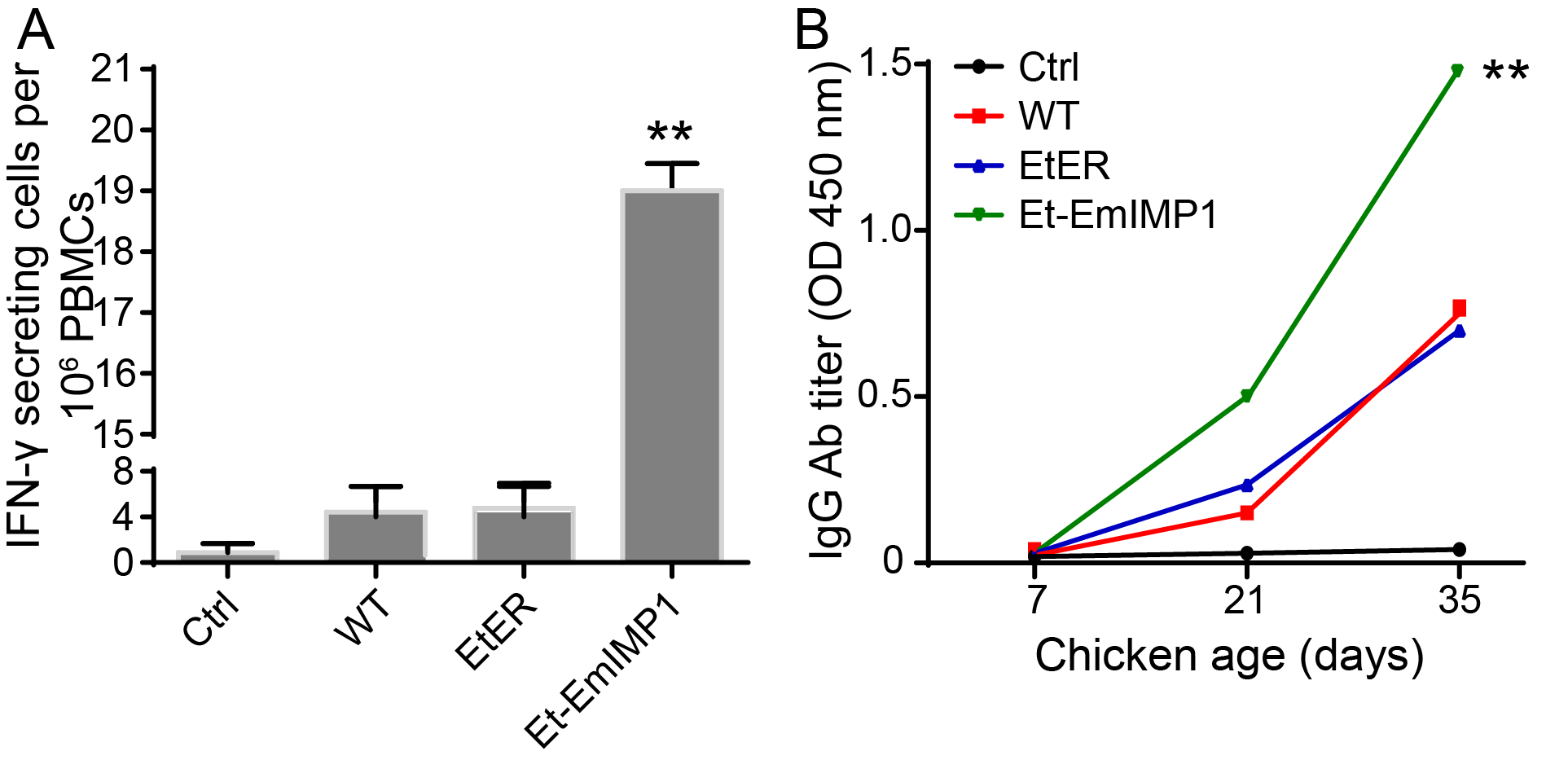

Supplement: Figure S1 — Vaccination with Et-EmIMP1 elicited EmIMP1-specific immune responses. (A) Mean number of EmIMP1-specific IFN-γ secretion lymphocytes in peripheral blood mononuclear cells (PBMCs) in Et-EmIMP1 immunized chickens (n = 3). (B) EmIMP1-specific antibody titer increased after primary (21 days) and secondary (35 days) immunization with Et-EmIMP1 significantly higher than its wild type. Each value represents the mean ± SD of three birds. *p < 0.05; **p < 0.01. [file Image_1.tif]

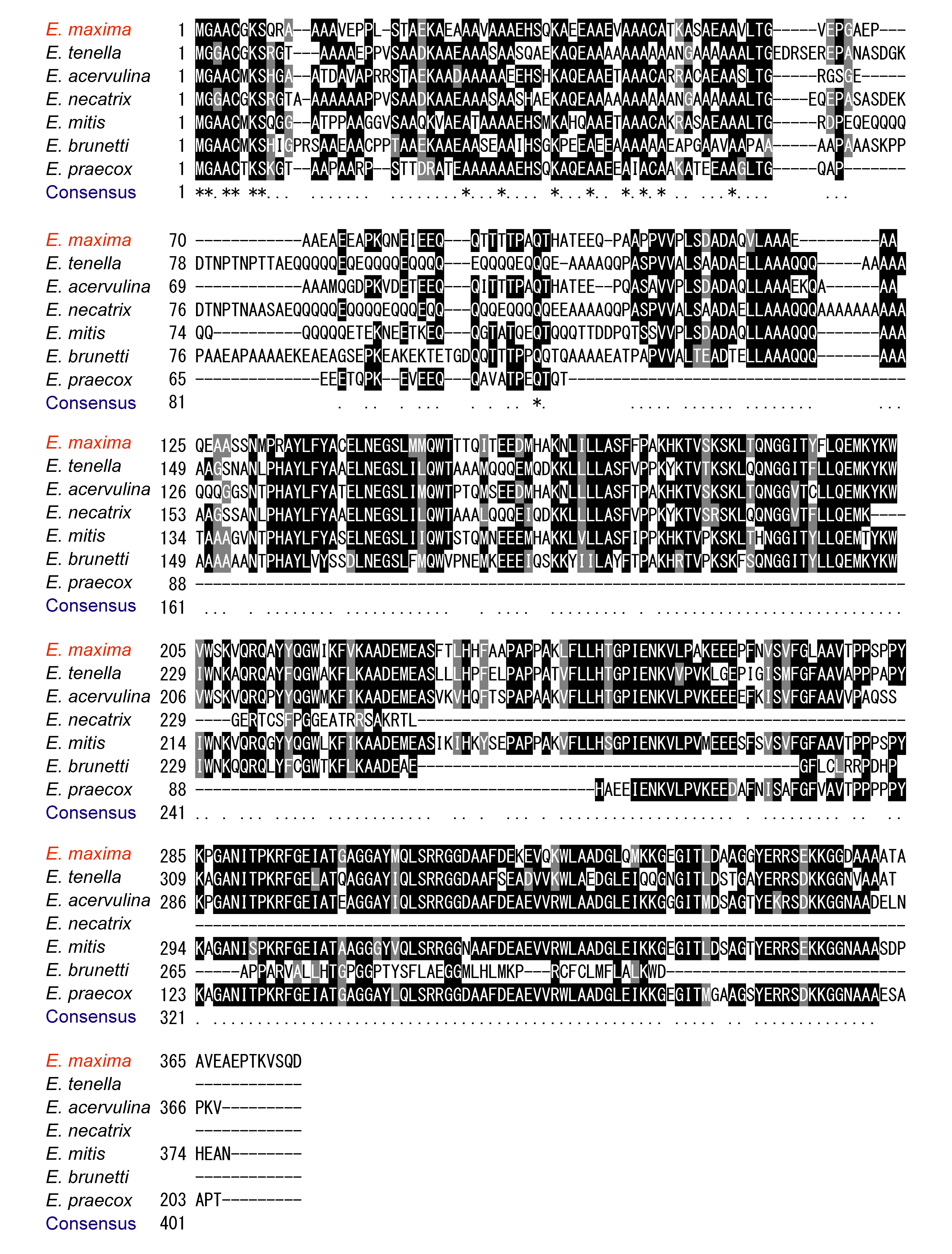

Supplement: Figure S2 — Multiple alignment of immune mapped protein 1 (IMP1) of chicken coccidia. Immune mapped protein 1 of E. maxima (EmIMP1)’s gene number is given in the Section “Materials and Methods.” GeneDB’s gene numbers of IMP1 of Eimeria tenella, Eimeria acervulina, Eimeria necatrix, Eimeria mitis, Eimeria brunette, and Eimeria praecox were ETH_00030475, EAH_00011210, ENH_00036180, EMH_0033960, EBH_0052010, and EPH_0045280, respectively. [file Image_2.tif]
