# Supplementary material for: A Novel Vaccine Delivery Model of the Apicomplexan Eimeria tenella Expressing Eimeria maxima Antigen Protects Chickens against Infection of the Two Parasites
Source: Front Immunol. 2018 Jan 10;8:1982. doi: 10.3389/fimmu.2017.01982 (PMC5767589; doi:10.3389/fimmu.2017.01982)
Supplement: Supplementary file 3 [file Table_1.docx]

**Table S1. Primers used in this study.**

| Primer name | Primer sequence (5’to 3’) | Notice |
| --- | --- | --- |
| IMP1-F | CATATGATGGGGGCCGCTTGCGGGAAAT | Nde I |
| IMP1-R1 | ACCGGTATCTTGCGACACTTTAGTGG | Age I |
| IMP1-R2 | GTGTCGCAAGATACCGGTGTCATCCTTGTA | Age I |
| IMP1-R3 | CCGCGG*TCA***CTTATCGTCGTCATCCTTGTA**ACCGGT | Sac II |

Bold-type letter: nucleic acid sequence of flag tag.

Italic letter: stop codon.
