# Supplementary material for: A Novel Vaccine Delivery Model of the Apicomplexan Eimeria tenella Expressing Eimeria maxima Antigen Protects Chickens against Infection of the Two Parasites
Source: Front Immunol. 2018 Jan 10;8:1982. doi: 10.3389/fimmu.2017.01982 (PMC5767589; doi:10.3389/fimmu.2017.01982)
Supplement: Supplementary file 4 [file Table_2.docx]

**Table S2. Stably transfected *Eimeria* parasite selection based on EYFP expression.**

| Transgenic parasite | Generations | % EYFP expression | Selection strategy |
| --- | --- | --- | --- |
| Et-EmIMP1 | 1 | 29.8 | Drug |
|  | 2 | 79.2 | Drug + FACS |
|  | 3 | 94.1 | Drug + FACS |
|  | 4 | 95.2 | Drug |
|  | 5 | 94.3 | Drug |
|  | 6 | 94.7 | --- |
